# Continuous Productivity Improvement Using IoE Data for Fault Monitoring: An Automotive Parts Production Line Case Study

**DOI:** 10.3390/s21217366

**Published:** 2021-11-05

**Authors:** Yuchang Won, Seunghyeon Kim, Kyung-Joon Park, Yongsoon Eun

**Affiliations:** Department of Information and Communication Engineering, Daegu Gyeongbuk Institute of Science and Technology, Daegu 42988, Korea; yuchang@dgist.ac.kr (Y.W.); seu7704@dgist.ac.kr (S.K.); kjp@dgist.ac.kr (K.-J.P.)

**Keywords:** internet of everything, production systems engineering, continuous productivity improvement, smart factory, fault monitoring data

## Abstract

This paper presents a case study of continuous productivity improvement of an automotive parts production line using Internet of Everything (IoE) data for fault monitoring. Continuous productivity improvement denotes an iterative process of analyzing and updating the production line configuration for productivity improvement based on measured data. Analysis for continuous improvement of a production system requires a set of data (machine uptime, downtime, cycle-time) that are not typically monitored by a conventional fault monitoring system. Although productivity improvement is a critical aspect for a manufacturing site, not many production systems are equipped with a dedicated data recording system towards continuous improvement. In this paper, we study the problem of how to derive the dataset required for continuous improvement from the measurement by a conventional fault monitoring system. In particular, we provide a case study of an automotive parts production line. Based on the data measured by the existing fault monitoring system, we model the production system and derive the dataset required for continuous improvement. Our approach provides the expected amount of improvement to operation managers in a numerical manner to help them make a decision on whether they should modify the line configuration or not.

## 1. Introduction

Collecting operation data from production systems in the factory floor has been a critical task to maintain the system operation and productivity. Most existing data collection systems are intended to monitor faults that are occurring from various machines in the system. Some are automated to raise alarms in ab-normalcy [1], and others rely on manual data collection and feed them to tools such as statistical process control [2]. Recently, thanks to the rapid advances of the Internet of Everything (IoE) technology, automated data collections are receiving a great deal of attention in the era of smart factory and Industry 4.0 with visions of using them beyond the fault detection and isolation: for preventive maintenance, job scheduling, productivity improvement, and various optimization [3,4]. Indeed, ref. [5] proposes to use an IoT-based architecture that collects information regarding key performance indicators to improve productivity, refs. [6,7,8] propose to build a digital twin for the production systems for multi-purpose optimization, ref. [9] suggests a smart factory framework, which has a cloud-assisted and self-organized structure to produce customized products in a real-time manner, and [10] suggests an IoT-based supply chain management system that tracks locations of goods to help managers check the status of a supply chain and its dependencies.

An important issue that can be addressed using the infrastructure of IoE enabled smart factory is the continuous improvement of a production line [11]. The continuous improvement is a major tool for production systems management, where projects are designed to improve productivity of the production systems. Specifically, continuous improvement projects involve bottleneck identification and elimination by allocating additional resources in order to achieve higher productivity in an efficient manner. In addition, analysis for the continuous improvement requires the capability of quantifying the improvement if characteristics of the bottleneck are changed. Existing studies [12] for methods of bottleneck identification and analysis for continuous improvement projects are based on measurement data, such as cycle-time (average time for a machine to finish a task), uptime (average time for a machine to be up, i.e., operational), downtime (average time for a machine to be down, i.e., not operational), and buffer capacity.

We point out that hardly any manufacturing facilities has dedicated IoE devices for direct measurement of cycle-time, uptime, and downtime for the continuous improvement while many facilities have basic fault monitoring systems. Unfortunately, cycle-time, uptime, and downtime are not directly available from fault monitoring systems. For example, the fault monitoring system in [13] represents machine states as ‘processing’, ‘inspection’, and ‘manual operation’ and that of a microfluidic device manufacturing line [14] categorizes machine states as ‘no operation’, ‘idling’, and ‘operating’. A monitoring system in an automotive part production line that is used for a case study in this work categorizes machine states as ‘working’, ‘idling’, ‘complete’, and ‘alarm’. Clearly, extracting cycle-time, uptime, and downtime of each machine from the mentioned fault monitoring data are not at all straightforward. Although many manufacturing facilities are installing IoE devices for data collection under the initiative of smart factory and Industry 4.0, the new devices are still installed with the main purpose of monitoring faults [15].

A method of using the data from the existing fault monitoring IoE systems for the purpose of the continuous improvement would save time and resource for the manufacturing facilities: new installation is not necessary which may avoid stopping the production for the installation. Thus, in this paper, we present a case study where the continuous improvement of an automotive parts production system is addressed using the data from a fault monitoring system. As mentioned earlier, the dataset necessary for the continuous improvement (i.e., uptime, downtime, and cycle-time) are not directly available from the fault monitoring systems. Therefore, we study the problem of how to derive the dataset of uptime, downtime, and cycle-time for the continuous improvement from the existing fault monitoring data.

In order to model and analyze production systems, many approaches and frameworks are available as reviewed in [16]. In this work, as a main tool for productivity analysis, we use the theory of production systems engineering (PSE) [11] due to three distinct advantages: evaluation of various performance metrics is possible for production systems; convergence of the numerical algorithm in PSE is analytically proven; and it has been applied to various actual manufacturing systems.

The theory of PSE models a production line with machines and buffers, where machines are characterized by uptime, downtime, and cycle-time. The aggregation algorithm approximates the model of the serial production line as one virtual machine by aggregating the consecutive two machines and one buffer, recursively. Using this aggregation algorithm, the theory of PSE provides the foundation of modeling production systems and predicting performance characteristics, such as throughput, transient [17,18], lean buffering [19], lead time [20], bottleneck machine, and bottleneck buffer [12].

The aggregation algorithm is analytically proven to converge [11]. This is a significant advantage compared to other methods. For instance, the convergence of the ADDX algorithm used in the decomposition approach [21] is not analytically guaranteed.

Various productivity analysis cases based on the theory of PSE have been reported (an automotive paint shop line [22], a lighting equipment assembly line [23], a ham shaving and packaging line [24], and a gear assembly line in a motorcycle powertrain manufacturing plant [25]).

Finally, several major manufacturing companies appear to have in-house tools and methods, but these are not publicly available. Discrete event simulations could be an alternative approach, but are computationally much heavier than the methods PSE provide, especially, when number of machines and capacity of buffers are large.

Our case study pertains to an automotive part production line. The line has a fault monitoring system that observes the status of all the machines in the production system. We present a method of extracting uptime, downtime, cycle-time from the fault monitoring data. Then, based on PSE, we model the production line with appropriate parameters. In turn, we use this model to address continuous improvement projects under various scenarios.

The main contributions of this paper are as follows:

- We propose a concept of using existing fault monitoring data for the purpose of continuous improvement of production systems;
- We present a case study using an automotive parts production line;
- We develop a mathematical model of the line that predicts key performance characteristics, such as throughput, lead time, bottleneck machine, and bottleneck buffer;
- Based on the model, we develop a continuous improvement scenario that leads to up to 10% of productivity improvement.

The outline of the rest of the paper is as follows. Section 2 describes the production line we consider. Additionally, description of the fault monitoring data are given. In Section 3, we discuss the challenges why fault monitoring data are not directly transferable to uptime, downtime, and cycle-time. Then, we introduce a method of conversion for this particular production line considered. Based on the estimated parameters, we create a model and analyze the production line with the theory of PSE in Section 4. Section 5 shows the continuous improvement results in a few scenarios. Finally, conclusions are presented in Section 6.

## 2. Automotive Parts Production Line and Fault Monitoring Data

### 2.1. Production Line

**Figure 1:**
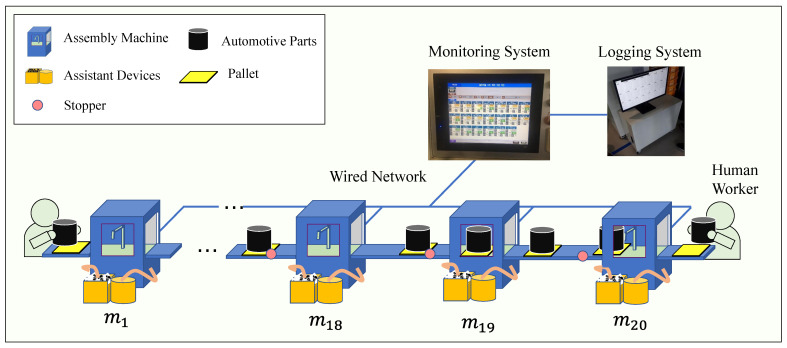
Automotive parts production line with fault monitoring and logging system.

The plant covered in this paper is an automotive parts assembly line from a tier-1 vendor for a world top-5 motor company. We consider an automotive parts production line whose simplified illustration is shown in Figure 1. The line comprises 20 assembly machines connected serially. We refer to each machine by $m_{i}$, i=1,2,⋯,20 in the order of the part flow in the production system. The machines $m_{1}$ and $m_{20}$ are semi-automatic, i.e., operated by human workers and the rest are automatic. Machines assemble sub parts and inspect defectiveness of products. Sub parts and assembled parts are moving on pallets in the production line. Each pallet is identified by an RFID, the reader of which is installed on all machines in the line. There are various assisting devices to some machines that provide necessary materials (screw, lubricants, etc.). Semi-assembled products are placed on a pallet and moved to the next machine.

All machines are connected by the pallet conveyor system. The pallet conveyor system transfers pallets from $m_{1}$ to $m_{19}$. After passing $m_{19}$, pallets return to the first machine. The total number of pallets is 40. The pallet conveyor system has stoppers to block the pallet from getting into the machine. The stopper is in front of the entrance of the machine as shown in Figure 1. All pallets stop at the stopper once. If the machine is full, a pallet waits at the stopper until the machine is empty. If not, then the pallet goes into the machine.

Among 20 machines, $m_{15}$, $m_{16}$ are identical, and $m_{17}$, $m_{18}$ are also identical to each other. This is because the task by $m_{15}$ and $m_{17}$ are taking about twice as much time as the other tasks. Hence, two identical machines are allocated for the job in order to speed up the process. Their operations are as follows. If $m_{15}$ operate only on the parts delivered by even numbered (pallet RFID) pallets. It passes the odd numbered pallets to $m_{16}$. The other pair $m_{17}$ and $m_{18}$ are operated in a similar manner.

The machines $m_{2}$, $m_{6}$, $m_{9}$ operate with block before service (BBS) and the other machines operate with block after service (BAS), where BAS and BBS are rules for interacting between machines and buffers. When the downstream buffer of a machine is full, the machine should stop producing. In this situation, the machine with the BAS rule produces one product and keeps it inside the machine. On the other hand, the machine with the BBS rule does not produce and leaves its inside space empty [11].

The line produces a total of 52 types of products. The machines need to change their settings whenever the types of products change. It takes time to change the settings, therefore the company operates the line with a batch production rule to reduce the process change time where the batch refers to a group of products of the same type. As the machines are differently operated by the product type, the throughput of the line may also be different product types.

Figure 1 also shows a fault monitoring system. Every machine transfers its operation data to the fault monitoring system at every second. The monitoring system represents all machine’s states right after receiving the operation data from each machine. The fault monitoring system in this manufacturing facility *does not record data* perhaps because it is designed only for raising alarm at faults. We develop the logging system which takes all data from the monitoring system and write the data to a file by the hour.

The machines rarely produce defective products. Nevertheless, the line has the capability built in to deal with the defective parts. The defective products are not removed from the production line immediately. If a machine generates a defective product, then the machine informs to the monitoring system. After that, the monitoring system transmits the serial number of the defective product to the downstream machines so that the downstream machines just pass the defective product until $m_{11}$ or $m_{19}$, which are inspection machines. The inspection machines eliminate defective parts into their basket.

The production line operates for 24 h with several break times.

### 2.2. Fault Monitoring Data

Using the logging system described in the previous subsection, we obtained fault monitoring data for five months in 2019. The fault monitoring data contain machine state, product state, processing time, serial number, and logging time. An example of the data is shown in Table 1, where ‘Time’, ‘Type’, ‘M State’, ‘P State’, ‘SN’, and ‘PT’ refer to ‘Logging Time’, ‘Product Type’, ‘Machine State’, ‘Product State’, ‘Serial Number’, and ‘Processing Time’, respectively.

The item ‘Machine State’ in the operation data indicates the operate state of the machine at a specific time. The machine reports its state by ‘Idling’, ‘Working’, ‘Complete’, or ‘Alarm’. The detailed description of ‘Machine State’ are as follows:

- A machine reports ‘Idling’ when the inside of the machine is empty;
- A machine reports ‘Working’ when the machine does assembling, inspecting, or other actions for producing products;
- A machine reports ‘Complete’ after finishing production processes, and sustains ‘Complete’ until its inside becomes empty;
- A machine reports ‘Alarm’ if the machine is in a breakdown.

The item ‘Product State’ represents the inspection results of the defectiveness of the assembled product. The machine informs the state of the product as ‘Stand by’, ‘OK’, and ‘NO’.

- A machine represents ‘Product State’ as ‘Stand by’ after the machine takes a product;
- After finishing the inspection, the machine reports ‘Product State’ as ‘OK’ if there is no problem with the product;
- If the machine identifies defective parts, then the machine reports ‘Product State’ as ‘NO’.

The rest of the data include ‘Serial Number’, ‘Processing Time’, ‘Time’, and ‘Type’. The item ‘Serial Number’ refers to the sequence of products during a day. The monitoring system initiates ‘Serial Number’ to 1 at midnight. The monitoring system sequentially assigns ‘Serial Number’ to pallets by reading RFID at the first machine, and removes it at $m_{19}$. The item ‘Processing Time’ indicates how long the pallet stays inside the machine for producing. The item ‘Time’ indicates when the log is recorded, and ‘Type’ represents a product type in the first machine.

## 3. Obtaining Uptime, Downtime, Cycle-Time from the Fault Monitoring Data

Obviously, the data shown in Table 1 are not in a form from which the uptime, downtime, and cycle-time of each machine are obtained in a straightforward manner. As we pointed out in the introduction, this is due to that the fault monitoring data collection are not intended for continuous productivity improvement. This difficulty of mismatch is dealt with in detail in Section 3.2.

**Figure 2:**
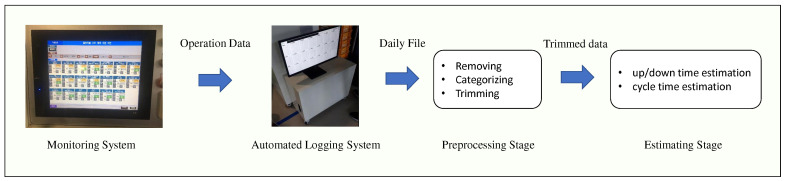
Parameter estimation procedure.

Additionally, as alluded to in Section 2.2, uptime, downtime, and cycle-time may be different by the types of the product. Hence, the first step is to isolate the time segment where a given product type is produced. Therefore, we propose a parameter estimation method consisting of two stages, a preprocessing stage and an estimating stage. The preprocessing stage is trimming the fault monitoring data: removing the break time from the log, classifying the product types, and removing the logs that corresponds to initial transient state. The parameters, uptime, downtime, and cycle-time, are estimated by the second stage based on the trimmed data. The entire procedure for estimating the parameters are simplified in Figure 2.

### 3.1. Preprocessing Stage

**Figure 3:**
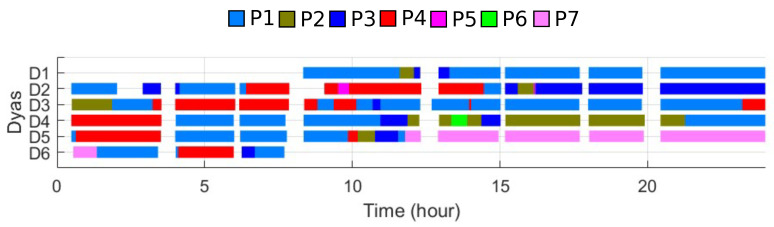
Daily operations (determined by the idle time of $m_{1}$). Different part types are shown by different colors. Data from Month 4 in 2019. The line produces multiple types of parts in a day. Most breaks are planned, but others exist due to unexpected (either workers or machines) situations.

Figure 3 shows typical daily operation of the production line for a week in the Month 4 of 2019. This snapshot of the operation data is obtained as follows. First, break time had to be determined from the logs. For this purpose, we use the ‘SN’ of $m_{1}$: if ‘SN’ of $m_{1}$ does not change for more than 10 min, we determine that the production line is not operational (break time for the workers). The color of the bar represents different product types. This is determined by the ‘Type’ data in $m_{1}$ from the fault monitoring dataset. We point out that Figure 3 is the result of preprocessing that identifies in automatic manner the break time and the types.

From Figure 3, one may use all the data segment with the same color to extract the cycle-time of each machine. However, for uptime, another aspect must be taken into account. When the machine is in transient state, total operation time may not be accurate, which affects the calculation of uptime (uptime is computed by subtracting downtime from the total operation time). Hence, we additionally remove the first portion of the data until the last machine completes five products. Therefore, we cut the data related to the first five products off in the fault monitoring data in order to generate trimmed data.

### 3.2. Estimating Stage

The purpose of this stage is to extract uptime, downtime, and cycle-time of individual machine (from $m_{1}$ tot $m_{20}$) for a given product type. Trimmed segments for a given product (same color in Figure 3) are used.

**Figure 4:**
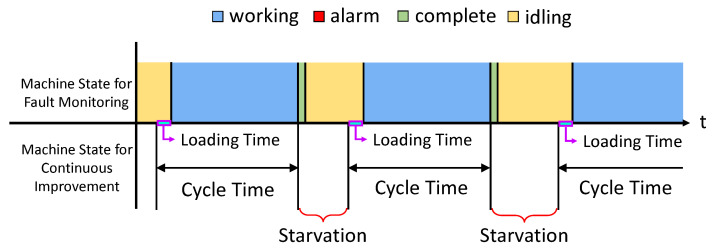
Machine state for fault monitoring (existing fault display system) and machine state for continuous improvement.

We first discuss how to obtain a cycle-time for each machine. The cycle-time is identified by searching ‘Idle’-‘Working’-‘Complete’ states sequence in the fault log. This is illustrated in Figure 4. It may appear that after find the sequence, use ‘Working’ state as one instantiation of the cycle-time may suffice. However, after observing the operation on the factory floor for an extended period of time, we realize that computing cycle-time in this manner may not be accurate: there is time, referred to as *loading time*, for a machine to load the product from the pallet. This portion must be included in the cycle-time, but it is included in the ‘Idle’ state according to the fault log. As shown in Figure 4, we extract the sequence in the log, then identify the duration of ‘Working’ and add to it the loading time to obtain a realization of the cycle-time.

**Figure 5:**
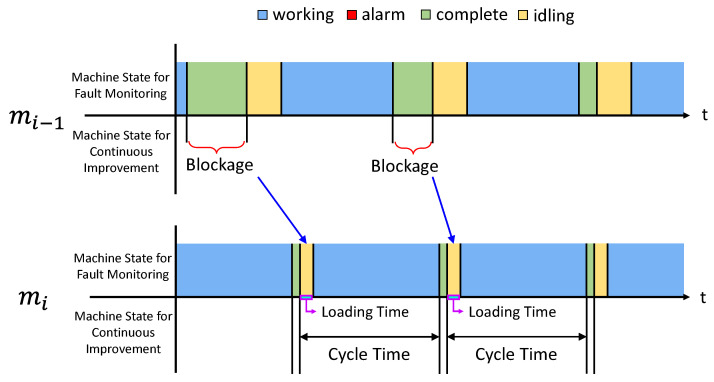
Machine state for fault monitoring and machine state for continuous improvement in blockage cases.

For this procedure to work, the loading time for each machine needs to be determined. As it turns out, we can identify the loading time from the log in a specific situation called blockage. Blockage means that a machine completes the task, but cannot move the part to the down stream buffer because the buffer is full. In order to identify the loading time of $m_{i}$, the blockage of $m_{i-1}$ has to be searched. The condition for this is to look for a prolonged ‘Complete’ state of $m_{i-1}$ (because $m_{i-1}$ cannot push the product out). When $m_{i-1}$ is in blockage, the upstream buffer for $m_{i}$ is full. Thus, $m_{i}$ takes the part right after it finishes the task on the previous part. This means the duration of the ‘Idle’ state in $m_{i}$ is equal to the loading time of the next machine. An illustration is given in Figure 5.

A code is written to identify for each machine the above described conditions. It results more than thousand cases for loading time, the average of which is used as ‘loading time’ for the machine.

**Figure 6:**
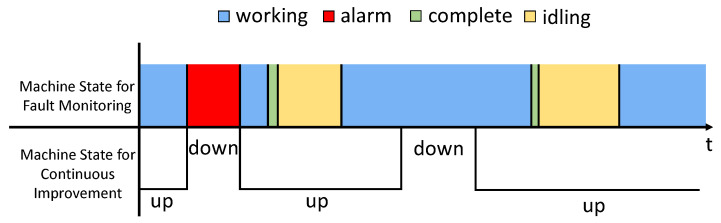
Machine state for fault monitoring and machine state for continuous improvement in down cases.

Next, we attend to the up and down time. From a continuous improvement analysis point of view (e.g., PSE analysis framework) each machine state is either up or down. Up state means that a machine is operational, and down state means that the machine is not operational. The time that a machine is waiting for a product to arrive (starvation), although the machine is not producing, is counting up towards a certain state that the machine is capable of producing. The time that a machine cannot produce due to the shortage of assembly parts (e.g., shortage of screws) although the machine is not out of order is counted toward down state. Obviously, this classification of up and down state does not match with machine state recorded in fault monitoring data. We illustrate this by Figure 6.

The first down state shown in Figure 6 matches with the ‘Alarm’ state (i.e., the machine is out of order). However, the second down state does not show at all in the log. This was due to the lack of assembly supplies. Uptime does not exactly align with ‘Working’ state either.

In the theory of PSE, downtime is the average amount of time a machine cannot produce, even if it is capable of producing. We observed two situations for this production system that corresponded to downtime of the machines. First is the breakdown of the machines. This is indicated by ‘Alarm’. The second is running out of additional assembly parts and materials (screws, lubricants, etc.) that are necessary for the assembly. The second case does not correspond to any state in fault monitoring data. We identify this by looking at abnormally long ‘Working’ state. Since we computed cycle-time earlier, the abnormally long means that it is longer than 1.5 times the cycle-time. The long working duration minus the cycle-time is counted toward down time. Again, a code is written to identify all such cases for each machine to determine down time.

Once the down time is obtained, uptime is computed by subtracting down time from a total operation time. The total operation time is computed from the trimmed data.

It must be pointed out that, although we discuss in detail the fault monitoring data of the production system considered in the case study, no generalization is given how to obtain cycle-time, uptime, and downtime from general fault monitoring data. For instance, the fault monitoring data of [13,14] would require algorithms different from those used in this work.

## 4. Modeling, Validation, Bottleneck Identification

### 4.1. Modeling Framework for Continuous Improvement

**Figure 7:**
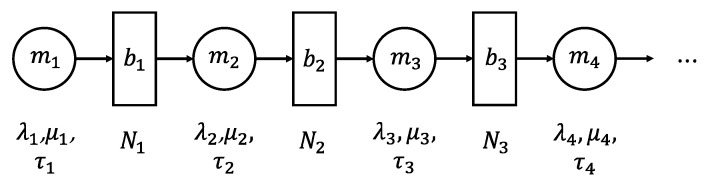
PSE model structure.

A very brief summary of [11] on the part relevant to this work is given here. In order to quantify the productivity, a production system has to be modeled. The model consists of serially connected machines and buffers. A machine is modeled by its cycle-time and reliability characteristics. The cycle-time denoted by τ. Reliability of each machine is modeled by probability distribution of the uptime. Here, uptime is modeled to be exponential distributed with λ. Then, λ is given by the reciprocal of the mean of uptimes. Similarly modeled is the downtime with a parameter μ set to the reciprocal of mean of the downtimes. Buffer capacity is given by a non-negative integers. An illustration is given in Figure 7. We refer to parameters of each machine by $\lambda_{i}$, $\mu_{i}$, and $\tau_{i}$, i={1,2,3,4,⋯} and the buffer capacity of each buffer by $N_{j}$, j={1,2,3,⋯}. Then, the theory of [11] provides methods of bottleneck identification and tools to analyze the model to obtain various performance metrics, such as throughput, work-in-process, lead-time, starvation, and blockage.

### 4.2. Structural Modeling of Production Line

The production line considered consists of serially connected 20 machines. This means there are up to 19 buffers between each of the machines. We fist calculate the buffer capacity. The capacity of the buffers is calculated with the velocity of the pallet, the size of the pallet, and the distance between the machines [11]. If the machine operates with the BAS rule, the buffer capacity should be increased by 1. If the machine runs under the BBS rule, the capacity remains the same. The calculated buffer capacities are in Table 2, where $b_{i}$ is *i*th buffer.

The capacities of the buffer $b_{2}$, $b_{6}$, and $b_{9}$ are modeled as zero. As a results, the machines, $m_{2}$, $m_{3}$, $m_{6}$, $m_{7}$, $m_{9}$, and $m_{10}$ can be simplified by aggregating them into $m_{2,3}^{agg}$, $m_{6,7}^{agg}$, and $m_{9,10}^{agg}$, respectively. In addition, the last machine $m_{20}$ is removed because $m_{20}$ rarely goes down; the cycle-time of $m_{20}$ is half of the cycle-time of $m_{19}$; and there is enough space between $m_{19}$ and $m_{20}$. Thus, $m_{20}$ as it is now does not affect the rest of the production line.

**Figure 8:**
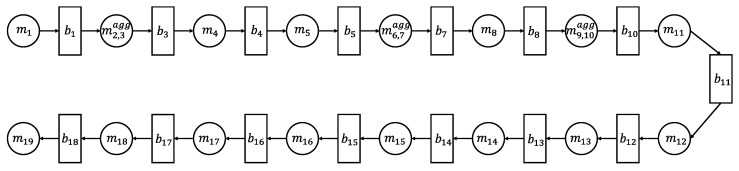
Simplified PSE model for automotive parts production line.

Based on the above simplification, we develop a serial PSE model with 16 machines as shown in Figure 8.

### 4.3. Machine Reliability Modeling

Table 3 shows the monthly normalized variation of the throughput for product type P1. For confidentiality reason, the data shown are normalized by the maximum. The monthly variations is about 2% which indicates that the underlying process does not seem to change over time. We build a model using the data from the latest month (Month 5) and use other sets for validation.

We use the parameters uptime, downtime, and cycle-time, estimated in Section 3.2 to calculate the parameters of each machine. Based on this parameters, we also calculate the parameters of the aggregated machines denoted by $\lambda_{i,i+1}^{agg}$, $\mu_{i,i+1}^{agg}$, $\tau_{i,i+1}^{agg}$ where i=2,6,9, as follows [11].

(1) $$\begin{array}{cc} \lambda_{i,i+1}^{agg} & =\frac{2}{\left(\frac{1}{\lambda_{i}}+\frac{1}{\mu_{i}}+\frac{1}{\lambda_{i+1}}+\frac{1}{\mu_{i+1}}\right)\left(e_{i}e_{i+1}\right)},\\ \mu_{i,i+1}^{agg} & =\frac{2}{\left(\frac{1}{\lambda_{i}}+\frac{1}{\mu_{i}}+\frac{1}{\lambda_{i+1}}+\frac{1}{\mu_{i+1}}\right)\left(1-e_{i}e_{i+1}\right)},\\ e_{i,i+1}^{agg} & =\frac{\mu_{i,i+1}^{agg}}{\lambda_{i,i+1}^{agg}+\mu_{i,i+1}^{agg}},\\ \tau_{i,i+1}^{agg} & =\max(\tau_{i},\tau_{i+1}), \end{array}$$

where

$$e_{i}=\frac{\mu_{i}}{\lambda_{i}+\mu_{i}}$$

Table 4 shows the model parameters of product type P1, $\lambda_{i}$, $\mu_{i}$, $e_{i}$, and $\tau_{i}$ based on Month 5 data ($\lambda_{i,i+1}^{agg}$, $\mu_{i,i+1}^{agg}$, $e_{i,i+1}^{agg}$, and $\tau_{i,i+1}^{agg}$ are also included). The units of $\lambda_{i}$ and $\mu_{i}$ are (1/minute), and in the case of $\tau_{i}$ is (second). Some machines rarely exhibit downtime. In this case we artificially use $\lambda_{i}=0.0004$ and $\mu_{i}=600$, which yields large enough uptime and almost no downtime. Based on the data we use asynchronous exponential line model type [11].

### 4.4. Model Validation

Using the recursive algorithm introduced in [11], we can calculate the throughput of the asynchronous exponential production line as follows.

(2) Throughput=TP(Λ,M,T,Θ),

where

$$\begin{array}{cc} \Lambda & =\{\lambda_{1},\lambda_{2,3}^{agg},\lambda_{4},\lambda_{5,6}^{agg},\lambda_{7},\lambda_{8},\lambda_{9,10}^{agg},\lambda_{11},\cdots ,\lambda_{19}\},\\ M & =\{\mu_{1},\mu_{2,3}^{agg},\mu_{4},\mu_{5,6}^{agg},\mu_{7},\mu_{8},\mu_{9,10}^{agg},\mu_{11},\cdots ,\mu_{19}\},\\ T & =\{\tau_{1},\tau_{2,3}^{agg},\tau_{4},\tau_{5,6}^{agg},\tau_{7},\tau_{8},\tau_{9,10}^{agg},\tau_{11},\cdots ,\tau_{19}\},\\ \Theta & =\{N_{1},N_{3},N_{4},N_{6},N_{7},N_{8},N_{10},N_{11},\cdots ,N_{19}\}. \end{array}$$

**Figure 9:**
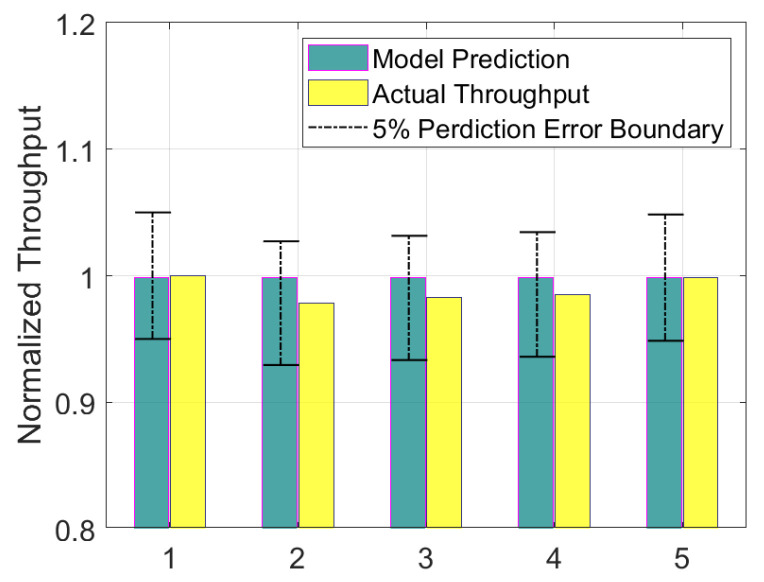
Comparison of actual throughput and model prediction.

Throughput prediction results are shown in Figure 9. Green and yellow bars represent model prediction throughput and actual throughput, respectively. The black dash-dotted line shows 5% error boundary of the actual throughput. The error is a percentage error defined as follows.

(3) $$Error\left(\%\right)=\frac{\left|Model-Actual\right|}{Actual}\times 100$$

where Model means model prediction value and Actual means actual throughput of the production line.

The model accuracy under the 5% error is acceptable in the field of manufacturing [11]. As shown in Figure 9, the model prediction values have an error of less than 5%, which indicate that the parameter estimation method presented in Section 3 are acceptable. We emphasize here that the monthly data used for the analysis came from the fault monitoring system (which is never intended for continuous improvement analysis). Judging from the accuracy, the work of converting the fault data to uptime, downtime, cycle-time appear to be highly effective.

### 4.5. Bottleneck Identification

To improve the performance of the production line, the bottleneck machine identification method is defined in [12] as follows.

**Definition** **1.** *Consider the asynchronous exponential line with M machines. Exponential machine $m_{i}$, i∈{1,⋯,M} is bottleneck if*

(4) $$\frac{\partial TP(\Lambda ,M,T,\Theta )}{\partial c_{i}}>\frac{\partial TP(\Lambda ,M,T,\Theta )}{\partial c_{j}},\;\forall j\neq i$$

*where $c_{i}=1/\tau_{i}$.*

A simple way to identify the bottleneck machine is introduced in [12], called the arrow method. The arrow method is an algorithm. The inputs of this algorithm are starvation and blockage, which can be measured from the actual production line or can be calculated by the PSE model. Let the starvation and blockage of the *i*th machine as $ST_{i}$ and $BL_{i}$. If $BL_{i}>ST_{i+1}$, assign the arrow from $m_{i}$ to $m_{i+1}$. In the opposite case, the arrow is also assigned oppositely.

Based on the assigned arrows, we can identify the bottleneck machine with the following bottleneck indicator [12].

- If two arrows converge into a single machine, the machine is bottleneck machine;
- If more than two arrows converge into multiple machines, the machines are all bottleneck machines. One machine, which has the largest severity value denoted by $S_{i}$, becomes the primary bottleneck machine, where the severity value is defined as
  (5) $$\begin{array}{cc} S_{i} & =|ST_{i+1}-BL_{i}|+|ST_{i}-BL_{i+1}|,\\ S_{1} & =|ST_{2}-BL_{1}|,\\ S_{M} & =|ST_{M-1}-BL_{M}|. \end{array}$$
- If all arrows are in the same direction, the bottleneck machine is located the end of the line. In case that the first machine emanates the arrow, the last machine is the bottleneck machine. In the other case, the first machine is the bottleneck machine.

**Figure 10:**
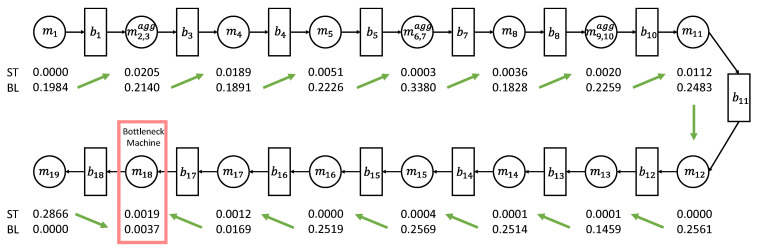
Bottleneck identification result.

The result of the arrow method is shown in Figure 10. The starvation and blockage are calculated by the PSE model. The machine $m_{18}$ is identified by the bottleneck machine.

## 5. Continuous Improvement Scenarios

### 5.1. Effect of Improving the Bottleneck

A scenario is considered that the bottleneck machine ($m_{18}$) is improved by reducing its cycle-time by 10%, i.e., 23.14 s to 20.83 s. The resulting throughput improvement, predicted by the developed model from Month 5 data, is 1.19%. Whether this amount is significant or not is the decision of the operation manager.

It turns out that if the cycle-time of $m_{18}$ is reduced by 1.6% (which is much less than 10%), the machine is not the bottleneck any more. The bottleneck has moved to $m_{17}$. Thus, more efficient strategy may be to reduce $\tau_{18}$ by 1.6%, and then reduce $\tau_{17}$ by the amount that it is not a bottleneck any more, and continue improving the subsequent bottlenecks. An implication of exercising this scenario is the production line is well ‘balanced’ or ‘close to optimal’ in the sense that improving a single machine does not yield a great amount of improvement of the whole.

### 5.2. Effect of Improving Multiple Machines

Since improving a single machine may not be an efficient strategy for this production line, here we consider the scenarios of improving multiple machines.

Notice that a portion of cycle-times is used for loading times for all machines. The loading time has its own reason to exist. It is, in fact, the time that takes each pallet to travel through repair area, which are from the stopper shown in Figure 1 to the entrance of downstream machine. The repair area ensures the repair space for workers so that the necessary repair or maintenance is completed in a short period of time. It means that the repair area reduces the downtime of the machine, but increase the cycle-time due to the loading time. Thus, eliminating the repair space will reduce the cycle-time, but it will increase the downtime.

**Figure 11:**
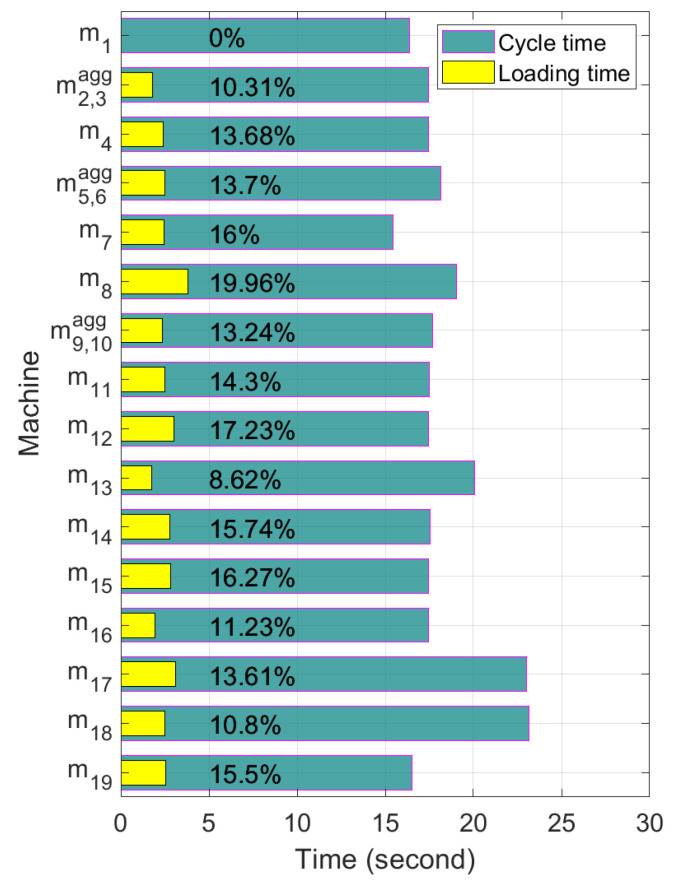
Proportion of loading time in cycle-time.

The estimated pallet loading times based on the operation data are shown in Table 5. Note that the pallet loading time occupies more than 10% of the cycle-time as shown in Figure 11. Thus, if we can remove the pallet loading time, then the cycle-time of machines could be reduced about 10%.

The scenario is that we remove the repair area of $m_{17}$ and $m_{18}$, hence reducing the cycle-times for each machine by the amount shown in Table 5. In consequence, downtime of each machine will increase. What is unknown here is the amount of increase in downtime if we remove the repair area (in order to reduce loading hence cycle-time). Thus, three cases are assumed for the amount: increase by 1 min, 5 min, and 10 min, uniformly for $m_{17}$ and $m_{18}$.

The results of the scenario are in Table 6. As one can see, the throughput of the line is increased by 9.41% in cases that the additional downtime is one minute. However, in cases that the additional downtime is 5 min or 10 min, the results show that the throughput is decreased by −0.31% and −10.64%, respectively. Thus, the throughput of this production system can be improved by removing the pallet loading time, if the additional downtime is less than 1 min. However, if the additional downtime is longer than 5 min, such modification yields no gain in the throughput.

In fact, removing repair areas of other combinations of the machines are also investigated. It turns out $m_{17}$ and $m_{18}$ is the best combination to improve the productivity.

## 6. Conclusions

Continuous improvement of the production line is one of the important issues of the manufacturing industry. Thanks to the advance of IoT technology, infrastructures to collect data are rapidly being developed. However, many data collection systems (especially, in middle-size companies) still focus on fault monitoring systems. The data of the fault monitoring system are not directly matched to the data required for continuous improvement project for productivity. Developing a new IoE enabled system dedicated for a continuous improvement project is time-consuming and incurs additional cost.

In this work, we propose a data processing method to use the conventional fault monitoring data for continuous improvement project. For an automotive part production line, a case study is presented where the dataset required for continuous improvement are derived from the dataset recorded for conventional fault monitoring system. Several conditions for this data conversion have been explained and illustrated. Then, using the converted dataset, the line is modeled with high accuracy based on the theory of productions systems engineering. Two improvement scenarios are considered using the model to quantify throughput improvement. In one of the scenarios, more than 9% productivity improvement is possible if the cycle-times are decreased for two machines out of 20 machines.

This study showcases a method of obtaining the information necessary for continuous improvement project from a legacy system. Extending the work to general fault monitoring systems, beyond the case study, would be a future work. We expect the results will be useful for manufacturing companies (especially middle-size) that are either building new IoE devices or seek additional benefits from the existing data collection systems.

## Figures and Tables

**Table 1:** Example of the operation data recorded by the logging system.

Time	Type	$m_{1}$				$m_{2}$				$m_{3}$	⋯
		M State	P State	SN	PT	M State	P State	SN	PT	M State	⋯
08:37:29	$P_{1}$	Working	Stand by	123	5.7	Complete	OK	120	13.5	Idling	⋯
08:37:30	$P_{1}$	Working	Stand by	123	6.7	Idling	OK	120	13.5	Idling	⋯
08:37:31	$P_{1}$	Working	OK	123	7.7	Idling	OK	120	13.5	Working	⋯
08:37:32	$P_{1}$	Working	OK	123	8.7	Idling	OK	120	13.5	Working	⋯
08:37:33	$P_{1}$	Working	OK	123	9.7	Working	Stand by	121	0.2	Working	⋯
08:37:34	$P_{1}$	Complete	OK	123	9.7	Working	Stand by	121	1.2	Working	⋯
08:37:35	$P_{1}$	Idling	OK	123	9.7	Working	Stand by	121	2.2	Working	⋯
08:37:36	$P_{1}$	Idling	OK	123	9.7	Working	Stand by	121	3.2	Alarm	⋯
08:37:37	$P_{1}$	Idling	OK	123	9.7	Working	Stand by	121	4.2	Alarm	⋯
08:37:38	$P_{1}$	Idling	OK	123	9.7	Working	OK	121	5.2	Alarm	⋯
08:37:39	$P_{1}$	Working	Stand by	124	0.2	Working	OK	121	6.2	Alarm	⋯
08:37:40	$P_{1}$	Working	Stand by	124	1.2	Working	OK	121	7.2	Alarm	⋯
08:37:41	$P_{1}$	Working	Stand by	124	2.2	Working	OK	121	8.2	Alarm	⋯
08:37:42	$P_{1}$	Working	Stand by	124	3.2	Working	OK	121	9.2	Alarm	⋯
08:37:43	$P_{1}$	Working	Stand by	124	4.2	Complete	OK	121	9.2	Alarm	⋯
08:37:44	$P_{1}$	Working	No	124	5.2	Complete	OK	121	9.2	Alarm	⋯
08:37:45	$P_{1}$	Working	No	124	6.2	Complete	OK	121	9.2	Alarm	⋯
⋮	⋮	⋮	⋮	⋮	⋮	⋮	⋮	⋮	⋮	⋮	⋱

**Table 2:** Buffer capacity of the production line.

Buffer	$b_{1}$	$b_{2}$	$b_{3}$	$b_{4}$	$b_{5}$
Capacity	1	0	1	1	1
Buffer	$b_{6}$	$b_{7}$	$b_{8}$	$b_{9}$	$b_{10}$
Capacity	0	1	1	0	1
Buffer	$b_{11}$	$b_{12}$	$b_{13}$	$b_{14}$	$b_{15}$
Capacity	1	2	2	2	1
Buffer	$b_{16}$	$b_{17}$	$b_{18}$		
Capacity	2	1	1		

**Table 3:** Monthly normalized throughput for product type P1.

	Month 1	Month 2	Month 3	Month 4	Month 5
Normalized Throughput	1	0.978	0.982	0.985	0.998

**Table 4:** Estimated parameters of product type P1 based on Month 5 data.

	$m_{1}$	$m_{2,3}^{\mathrm{agg}}$	$m_{4}$	$m_{5}$	$m_{6,7}^{\mathrm{agg}}$	$m_{8}$	$m_{9,10}^{\mathrm{agg}}$	$m_{11}$
$\lambda_{i}\;\left(\lambda_{i,i+1}^{agg}\right)$	0.5899	0.0265	0.0169	0.0019	0.0043	0.0095	0.0335	0.0004
$\mu_{i}\;\left(\mu_{i,i+1}^{agg}\right)$	5.0808	0.9443	1.7065	2.3875	0.8478	2.0906	1.5939	600.000
$e_{i}\;\left(e_{i,i+1}^{agg}\right)$	0.8960	0.9727	0.9902	0.9992	0.9949	0.9955	0.9794	1.0000
$\tau_{i}\;\left(\tau_{i,i+1}^{agg}\right)$	16.40	17.46	18.47	18.17	15.44	19.04	17.68	17.48
	$m_{12}$	$m_{13}$	$m_{14}$	$m_{15}$	$m_{16}$	$m_{17}$	$m_{18}$	$m_{19}$
$\lambda_{i}$	0.0032	0.0011	0.0074	0.0004	0.0100	0.0062	0.0184	0.0296
$\mu_{i}$	3.1400	2.3957	2.5368	600.0000	1.8715	1.7231	1.7805	2.7315
$e_{i}$	0.9990	0.9995	0.9971	1.0000	0.9947	0.9964	0.9898	0.9893
$\tau_{i}$	17.47	20.07	17.53	17.46	17.46	23.00	23.14	16.52

**Table 5:** Loading time of the production line.

Machine	$m_{1}$	$m_{2,3}^{agg}$	$m_{4}$	$m_{5}$
Loading Time	0	1.80	2.39	2.49
Machine	$m_{6,7}^{agg}$	$m_{8}$	$m_{9,10}^{agg}$	$m_{11}$
Loading Time	2.47	3.80	2.34	2.50
Machine	$m_{12}$	$m_{13}$	$m_{14}$	$m_{15}$
Loading Time	3.01	1.73	2.76	2.84
Machine	$m_{16}$	$m_{17}$	$m_{18}$	$m_{19}$
Loading Time	1.96	3.13	2.50	2.56

**Table 6:** Productivity improvement when loading times for $m_{17}$ $m_{18}$ are removed and downtimes are increased.

Increase in Downtime	1 min	5 min	10 min
Improvement (%)	9.41%	−0.31%	−10.64%

## Data Availability

Authors may not be able to provide the raw data due to confidentiality reasons with the partnered company.
